# Stress Effects on Mood, HPA Axis, and Autonomic Response: Comparison of Three Psychosocial Stress Paradigms

**DOI:** 10.1371/journal.pone.0113618

**Published:** 2014-12-12

**Authors:** Grace E. Giles, Caroline R. Mahoney, Tad T. Brunyé, Holly A. Taylor, Robin B. Kanarek

**Affiliations:** Department of Psychology, Tufts University, Medford, Massachusetts, United States of America; Technion - Israel Institute of Technology, Israel

## Abstract

Extensive experimental psychology research has attempted to parse the complex relationship between psychosocial stress, mood, cognitive performance, and physiological changes. To do so, it is necessary to have effective, validated methods to experimentally induce psychosocial stress. The Trier Social Stress Test (TSST) is the most commonly used method of experimentally inducing psychosocial stress, but it is resource intensive. Less resource intense psychosocial stress tasks include the Socially Evaluative Cold Pressor Task (SECPT) and a computerized mental arithmetic task (MAT). These tasks effectively produce a physiological and psychological stress response and have the benefits of requiring fewer experimenters and affording data collection from multiple participants simultaneously. The objective of this study was to compare the magnitude and duration of these three experimental psychosocial stress induction paradigms. On each of four separate days, participants completed either a control non-stressful task or one of the three experimental stressors: the TSST, SECPT, or MAT. We measured mood, working memory performance, salivary cortisol and alpha-amylase (AA), and heart rate. The TSST and SECPT exerted the most robust effects on mood and physiological measures. TSST effects were generally evident immediately post-stress as well as 10- and 20-minutes after stress cessation, whereas SECPT effects were generally limited to the duration of the stressor. The stress duration is a key determinant when planning a study that utilizes an experimental stressor, as researchers may be interested in collecting dependent measures prior to stress cessation. In this way, the TSST would allow the investigator a longer window to administer tasks of interest.

## Introduction

Psychological stress influences numerous psychological and physical processes in both healthy individuals and those with psychiatric disorders [1]–[3]. Stress is thought to influence mood [4], [5], memory [6], and decision-making [7]. The effects of psychological stress are physical as well, in that acute stress activates the hypothalamic-pituitary-adrenal (HPA) axis and sympathetic nervous system (SNS), producing elevations in cortisol, alpha-amylase (AA), and heart rate [8], [9].

Extensive experimental psychology research has attempted to parse the complex relationship between psychosocial stress, mood, cognitive performance, and physiological changes. For instance, understanding how acute stressors influence SNS response and performance on perceptual and cognitive tasks. Unfortunately much of this research proceeds without empirical understandings of how acute stress inductions may vary in their efficacy and duration. The present research was aimed at providing a first baseline understanding of how three commonly used stress inductions influence physiological, affective, and cognitive responding.

### Efficacy of experimental stress paradigms

The Trier Social Stress Test (TSST) [10] is the most commonly used method of experimentally inducing psychosocial stress. The TSST consists of three-parts, a 10-minute preparatory stage, 5-minute speech, and 5-minute mental arithmetic task, all in front of a panel of investigators, purportedly trained in analyzing nonverbal behavior [10]. Its socioevaluative and anticipatory components are thought to contribute to its efficacy as a stressor. The TSST is thought to be an effective method for inducing psychosocial stress, as it has repeatedly been found to increase anxiety, cortisol, and AA [11]–[13]. It also influences performance on a number of cognitive domains, including declarative memory (particularly for emotionally-arousing material), spatial, and working memory [14]–[16], as well as decision-making [7]. However, the TSST has a logistical limitation in that only one participant can be tested at a time and for each participant, numerous experimenters are required to staff the “panel.”

More recent and less resource intensive psychosocial stress tasks include the Socially Evaluative Cold Pressor Task (SECPT) [17]–[19] and computerized mental arithmetic task (MAT) [20], [21]. These tasks effectively produce a physiological and psychological stress response and have the benefit of requiring fewer experimenters and, in the case of the MAT, being able to run concurrent participants. However, it is unclear how these three tasks compare in terms of the magnitude and duration of the stress response.

Both cortisol and AA increase following onset of acute stressors, however whereas cortisol levels peak at 10–30 minutes after cessation of stressor and take approximately 90 minutes to return to baseline, AA levels peak 5–10 minutes post-stressor and take only 10–15 minutes to return to baseline [1], [22], [23]. Although multiple studies have assessed the relative time course of cortisol and AA levels before and up to one hour following acute stress [9], [22], [24], to our knowledge no study has assessed changes in mood and behavior beyond immediate post-stress induction. Therefore, it is not known how, exactly, the time course of changes in physiological indices of stress, such as cortisol and AA levels, relate to changes in mood and behavior.

In designing a study to analyze the effects of stress on a given cognitive function, it is necessary to know the duration of physiological and behavioral effects associated with the stressor (i.e. if stress-induced changes of the TSST are only significantly different from baseline for 30-minutes post-stress induction, the study should be designed such that any critical measures occur within that 30-minute window). Despite the practical importance of this knowledge, no study to date has assessed the differential magnitude and duration of experimental stress paradigms in a controlled, within-subjects design.

### Stress effects on working memory

Stress influences memory through the release of cortisol, which binds to glucocorticoid (GC) receptors located in the hippocampus and prefrontal cortex (PFC), making these two regions particularly vulnerable to stress [25]. Multiple studies have documented the influence of stress on hippocampal-dependent declarative memory, especially for emotionally arousing material [3]. However, the literature to date on prefrontal-dependent working memory is less consistent. Working memory refers to the limited capacity system in which information is temporarily stored, updated, and maintained [26]. While some studies have found that stress impairs working memory performance [15], [17], [18], [27], [28] others found no effects [17], [29].

We chose to examine stress-induced working memory changes using the N-Back Task, which challenges working memory by having participants monitor a series of briefly presented stimuli and decide on each trial if the current stimulus is the same as the one presented one, two or three trials before. The task emphasizes working memory monitoring and constant updating. The current design utilizes spatial cues at three levels of task load (1-back, 2-back, and 3-back) [30].

### The present study

The objective of this study was to compare the magnitude and duration of three experimental psychosocial stress induction paradigms. On each of four separate days, participants completed either a control non-stressful task or one of three experimental stressors: the TSST, SECPT, or MAT. We measured physiological response by collecting heart rate data throughout each test session. Participants first completed baseline salivary cortisol, AA, and affective state measures, followed by the stress induction. Following the stressor, participants completed three consecutive test blocks lasting 10 minutes each, consisting of affective questionnaires, a working memory task, and salivary cortisol and AA collection. We hypothesized that all experimental stressors would increase negative affect ratings, increase salivary cortisol and AA levels, and impair working memory performance. We also predicted that these effects would be most pronounced within conditions in the first test block (immediately following the stressor) and between conditions following the TSST and SECPT, which have the most pronounced socioevaluative components [31].

## Materials and Methods

### Participants

Twenty four undergraduate students (7 male; mean age 20. 63±1.91 years; mean BMI 20.91±2.89) participated for monetary compensation ($10 USD/hr). All participants were non-nicotine users and did not use prescription medication other than oral contraceptives. Exclusion criteria also included being pregnant or nursing, having a history of depression, anxiety disorders, panic attacks, cardiac disease, hypertension, or insomnia. Written informed consent was obtained, and all procedures were approved by the Tufts University Institutional Review Board.

### Design

We used a repeated measures design with four levels of our independent variable, Stress (TSST, SECPT, MAT, Control Task). Condition order was fully counterbalanced across participants.

### Profile of Mood States Questionnaire

The Profile of Mood States (POMS) is an inventory of subjective mood and arousal [32]. Participants rate a series of 65 mood related adjectives on a 5-point scale, using the response set of “how are you feeling right now?” The adjectives factor into six mood subscales (tension, depression, anger, vigor, fatigue, and confusion). The POMS is sensitive to a wide range of environmental factors; sleep loss, nutritional manipulations, and sub-clinical doses of various drugs [4], [33], [34].

### N-Back Task

The N-Back Task challenges working memory by having participants monitor a series of briefly presented stimuli and decide on each trial if the current stimulus is the same as the one presented one, two or three trials before. This task emphasizes working memory monitoring and constant updating. The current design utilizes a spatial N-Back Task, which involves monitoring object locations in different screen regions, each at three levels of task load: 1-back, 2-back, and 3-back [30]. Participants completed 57 trials within each load (total 171). Each stimulus was presented for 500 ms followed by a blank screen (2500 ms). Participants could respond either during the stimulus presentation or blank screen. Dependent measures include response time, hit rate, and sensitivity (d′).

### Arousal Measures

#### Salivary Cortisol and Alpha-Amylase

Saliva was collected for salivary cortisol and alpha-amylase (AA) analyses (biomarkers for arousal) using the SalivaBio Oral Swab (SOS) Method. Participants placed a swab under their tongue for 2 minutes. Swabs were placed into 1.8 ml plastic vials and immediately stored at -20°C (or colder) until assayed. Samples were analyzed in duplicate for cortisol and in singlet for AA in an independent laboratory (Salimetrics LLC, State College, Pennsylvania).

#### Heart rate

Heart rate data was collected using Equivital heart rate monitors. The monitor consisted of a transmitter worn against the skin and around the chest. The transmitters picked up and stored temporarily signals from the participant's heart and skin. The data was downloaded at the end of each experimental session. Participants were instructed on the proper placement of the heart rate strap and then asked to don the strap and sensor themselves. The experimenter then confirmed the signal.

### Stress Conditions

#### Trier Social Stress Test (TSST)

The TSST is a 20-minute psychosocial stress task consisting of 3 stages: (1) 10-minute preparatory stage, (2) 5-minute public speaking task, and (3) 5-minute mental arithmetic task [10]. In the first stage, participants were led into a conference room and introduced to a panel of three experimenters. They were given 10 minutes to prepare a 5-minute mock job-talk that would be videotaped and assessed for nonverbal behavior and voice frequency. In the second stage, participants delivered the 5-minute speech. If they ended in less than 5 minutes, they were asked to continue talking. In the third stage, participants completed a mental arithmetic task, in which they serially subtracted a prime number from a 4-digit number (e.g. 17 from 1223) and had to start over if they made a mistake.

#### Socially Evaluative Cold Pressor Test (SECPT)

**Figure 1:**
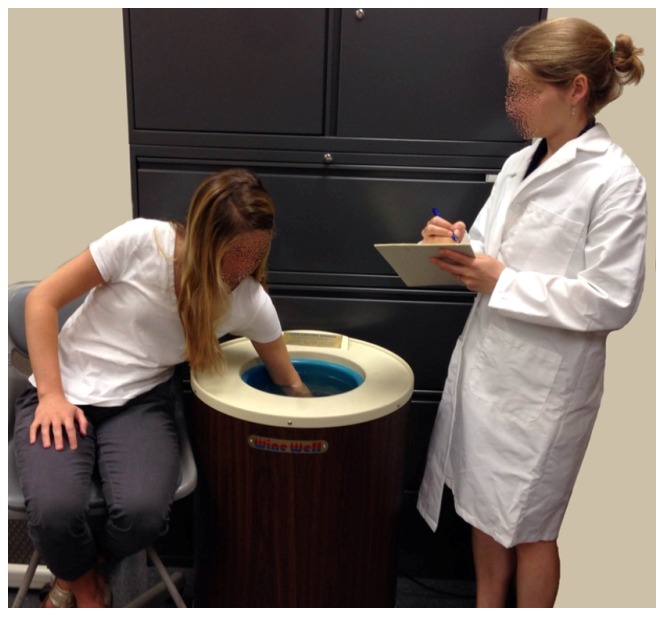
Socially Evaluative Cold Pressor Test (SECPT). Image represents the SECPT, in which participants immerse their arm, up to the elbow, in ice water (4°C) for up to 3 minutes, in front of a videocamera and experimenter.

In the SECPT, participants were led into a conference room. An experimenter explained that they would immerse their arm, up to the elbow, in ice water (4°C) for up to 3 minutes, and that they would be videotaped to later assess their facial expression (Fig. 1). Participants were told that they could remove their hand at any time, and were told when the 3 minutes had elapsed [19].

#### Arithmetic Task (MAT)

Participants performed a mental arithmetic for 20 minutes, similar to Kimura et al., 2006. Participants completed simple arithmetic problems presented one at a time on the computer monitor. Each problem appeared for 1000 ms and the participant had 1500 ms to respond using a standard numeric keypad. Feedback (correct/incorrect) was provided immediately following each response. Before beginning, participants were told that while actual performance varies on the task, the average performance of their peers for the given set of arithmetic problems is approximately 54% (as derived from pilot participants). Critically, to induce an acute psychosocial stress, the system adjusted problem difficulty to maintain an experimenter-set performance level below the “average student” performance level.

#### Control Stress Task

Participants completed a 20-minute neutral arithmetic task, similar to the stressful MAT, with the exception that the system adjusted problem difficulty to maintain an experimenter-set performance level approximately equal to “average student” performance of the participants' peers.

### Procedure

Participants completed five sessions on separate days: one practice session to become familiarized with the experimental procedure and tasks, and four test sessions corresponding to each Stress condition. During the practice session, participants completed screening materials and signed the informed consent. They were then familiarized with test procedures, including putting on the heart rate monitor, saliva collection and questionnaires. They then completed POMS. They then received instructions for the N-Back Task and completed practice trials. In addition, height and weight were taken. The practice and test sessions took place in the afternoon; beginning between 1300–1500 h. To control for potential effects of circadian rhythm, start time was consistent within each participant.

**Figure 2:**
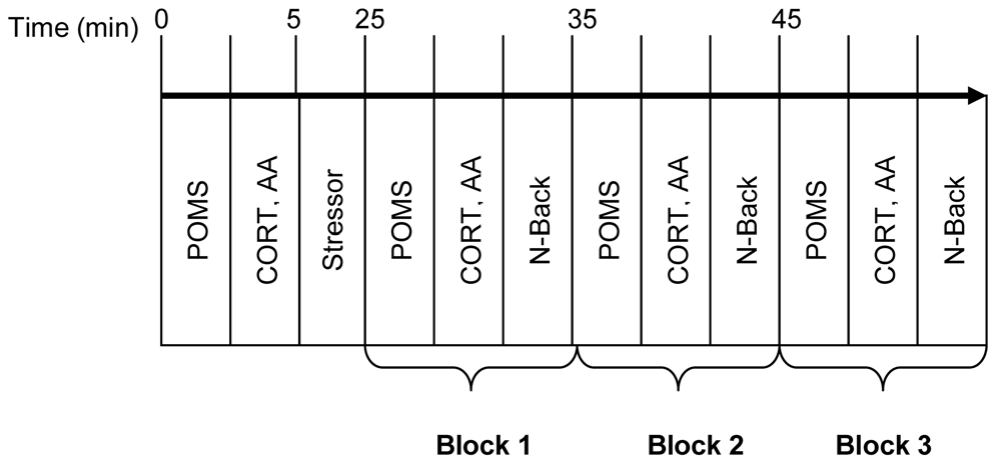
Schematic representation of the study schedule. During the study sessions, participants first completed baseline measures of the POMS and provided saliva samples for analysis of cortisol (CORT) and alpha-amylase (AA). They completed one of three experimental stressors, or the non-stressful control task. They then completed three blocks in succession, each consisting of the POMS, salivary measures, and a spatial N-Back task.

During test sessions, participants donned the heart rate strap and completed baseline measures of the POMS and saliva sample. Participants then completed the stress induction or non-stressful control task. After the stress induction they again completed the questionnaires and the N-Back Task, followed by a second saliva sample. The questionnaires, N-Back Task, and saliva sample constituted 1 block of the test battery. The participants completed 3 blocks in succession (see Fig. 2 for schematic of procedure).

### Statistics

The POMS and salivary measures were analyzed using an Analyses of Variance (ANOVA) with Stress condition (TSST, SECPT, MAT, Control Task) and Time (baseline and 0, 10-, and 20-minutes post-stress) as within-participants factors. Analysis of the N-Back Task was conducted using an ANOVA with Stress condition (TSST, SECPT, MAT, Control Task), Load (1-, 2-, and 3-Back) and Time (0-, 10-, and 20-minutes post-stress) as within-participants factors. Analyses of the heart rate data were conducted in the same manner with Time divided into the following 5 intervals: baseline, stress induction, 0-, 10-, and 20-minutes post-stress).

Salivary AA concentrations were first adjusted for salivary flow rate, calculated as 
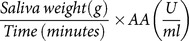
where Time equals two minutes. Adjustment for salivary flow rate is necessary given that the output of AA per unit time, not its concentration, is associated with the sympathetic stress response [35]. Because similar studies found that salivary cortisol and AA concentrations had skewed distributions [15], we tested for normality using the Lilliefors procedure. Cortisol and AA data showed a positively skewed distribution, and therefore were log-transformed. The ANOVAs were performed with the transformed data; for comprehensibility, only pre-transform data are presented in figures and tables. After removing outlying values, cortisol data reflects n  =  19 subjects and AA data reflects n =  23 subjects. In order to examine pre-stress differences, an additional ANOVA compared the four Stress conditions at baseline, i.e. before subjects were informed of the particular days' condition.

An effect was deemed statistically significant if the likelihood of its occurrence by chance was *p*<0.05. When sphericity was violated, Greenhouse–Geisser corrected *p*-values were used. When an ANOVA yielded a significant main effect, post-hoc tests using the Bonferroni correction were conducted. All statistical analyses were performed using SPSS 12.0.

## Results

### Stress Administration Order

The order of the Stress conditions was counterbalanced across participants to circumvent order effects. Four Stress conditions resulted in 24 possible orders, meaning each subjects was tested with a unique order. It was not feasible to fully assess order effects, given that each order would contain n  =  1. Nonetheless, all measures were subjected to analyses testing whether the First Condition completed influenced results. With two minor exceptions, no main effects or interactions were found with First Condition on the POMS, physiological measures, or N-Back task (all *p*s > 0.16). A significant Load x First Task interaction on N-Back hit rate *F*(2,40)  =  2.417, *p* <0.05 showed that response time was progressively higher as load increased when the SECPT, MAT and Control Tasks were administered first (*p*s <0.01), but not when the TSST was administered first (*p* > 0.16). Further, a marginal Stress Condition x First Task interaction on heart rate *F*(9,60)  =  1.768, *p* <0.1 was found but did not yield any significant effects on follow up tests (all *p*s >.11).

### Profile of Mood States (POMS)

The six mood subscales of the POMS were analyzed separately: tension, depression, anger, vigor, fatigue, and confusion, as well as total mood disturbance [32]. No effects were found for anger or depression. Analysis of the tension subscale revealed a main effect of Time *F*(3,69)  =  7.816, *p*<0.01 in which feelings of tension were significantly higher than baseline immediately post-stress and significantly lower than baseline at 10- and 20-minutes post-stress. A Stress x Time interaction *F*(9,207)  =  5.074, *p*<0.01 revealed that feelings of tension were significantly higher than baseline at all time points following the TSST *F*(3,69)  =  12.739, *p*<0.01, but were not significantly different following the other three tasks (Table 1). Analyses comparing the stress tasks to the control tasks showed that relative to the control task, feelings of tension were higher following the TSST immediately post-stress only *F*(3,69) = 6.998, *p*<0.01, but no effects were found for the SECPT, MAT or other time points.

**Table 1:** Profile of Mood States (POMS) as a function of stress (means and SE) at baseline and 0-, 10- and 20-minutes post-stress.

Adjective	Time	TSST		SECPT		Math		Control	
Tension	Baseline	1.17	(1.07)	1.79	(1.05)	2.17	(1.28)	2.71	(1.18)
	0-min	7.13**	(1.60)	2.21	(0.88)	4.21	(1.11)	3.38	(1.05)
	10-min	3.79**	(1.37)	2.33	(1.11)	2.71	(1.08)	4.13	(1.35)
	20-min	4.08**	(1.35)	2.50	(1.04)	2.71	(1.03)	3.96	(1.17)
Depression	Baseline	15.33	(.630)	3.04	(0.97)	12.96	(0.67)	5.33	(1.47)
	0-min	11.96	(1.440)	2.96	(0.92)	10.71	(0.86)	3.58	(1.05)
	10-min	10.21	(1.132)	5.17	(1.98)	9.46	(1.01)	5.42	(1.71)
	20-min	10.17	(1.283)	4.92	(1.58)	9.21	(0.88)	4.75	(1.67)
Anger	Baseline	3.33	(1.13)	2.25	(0.63)	4.75	(0.79)	3.50	(1.04)
	0-min	3.58	(1.61)	2.79	(0.93)	6.13	(1.01)	3.67	(1.17)
	10-min	4.38	(1.46)	3.92	(1.61)	5.67	(0.81)	4.88	(1.58)
	20-min	5.17	(1.33)	3.38	(1.27)	5.88	(0.97)	5.08	(1.53)
Vigor	Baseline	1.33	(1.32)	14.21	(0.92)	1.13	(1.48)	11.17	(1.18)
	0-min	2.29	(1.52)	13.38	(1.04)	2.33	(1.52)	10.75	(1.29)
	10-min	2.38	(1.49)	11.04	(1.31)	2.13	(1.44)	9.58	(1.38)
	20-min	2.92	(1.40)	9.79	(1.18)	2.63	(1.43)	9.13	(1.46)
Fatigue	Baseline	−4.75	(1.12)	3.58	(0.93)	−0.21	(1.27)	6.67	(1.21)
	0-min	10.25**	(1.01)	3.38	(0.82)	9.63*	(1.42)	5.17	(0.96)
	10-min	8.71**	(1.23)	4.29	(1.17)	7.83*	(1.41)	7.00	(1.28)
	20-min	11.04**	(1.33)	5.75	(1.32)	9.00*	(1.29)	7.21	(1.29)
Confusion	Baseline	1.17	(0.98)	1.00	(0.79)	2.17	(0.85)	2.63	(0.91)
	0-min	7.13**	(0.95)	1.42	(0.90)	4.21	(1.02)	1.63	(0.84)
	10-min	3.79**	(1.03)	2.54	(1.21)	2.71	(0.90)	2.17	(0.97)
	20-min	4.08**	(1.02)	3.00	(1.18)	2.71	(0.98)	2.58	(0.85)
Total Mood	Baseline	15.33	(4.68)	−2.54	(3.89)	12.96	(5.03)	9.67	(5.65)
	0-min	11.96	(6.41)	−0.62	(4.27)	10.71	(5.52)	6.67	(4.88)
	10-min	10.21	(5.94)	7.21	(6.86)	9.46	(5.36)	14.21	(6.42)
	20-min	10.17	(6.13)	9.75	(5.81)	9.21	(5.54)	14.25	(6.08)

*p<0.05, **p<0.01, levels of significance relative to baseline in stress x time interactions. Additionally, we found a main effect of the type of stress on the fatigue subscale, in which feelings of fatigue were lower during the TSST and SECPT than control condition *p*<0.05.

Analysis of the vigor subscale revealed a main effect of Time *F*(3,69)  =  21.386, *p*<0.01, in which vigor was significantly higher at baseline than all other time points.

Analysis of the fatigue subscale revealed a main effect of Stress *F*(3,69)  =  3.869 *p*<0.05 in which feelings of fatigue were significantly lower during the TSST and SECPT than the control condition. A main effect of Time *F*(3,69)  =  5.043, *p*<0.01 indicated that fatigue was higher 20-minute post-stress than at baseline.

Analysis of the confusion subscale revealed a main effect of Time *F*(3,69)  =  3.617, *p*<0.05, in which feelings of confusion were significantly higher 20-minutes post-stress than at baseline.

Analysis of the total mood disturbance subscale revealed main effect of Time *F*(3,69)  =  8.694, *p*<0.01 in which total mood disturbance was significantly higher than baseline at all other time points, and a Stress x Time interaction *F*(9,207)  =  3.287, *p*<0.05, in which total mood disturbance was higher than baseline at all three time points following the TSST *F*(3,69)  =  7.880 *p*<0.01 and MAT *F*(3,69)  =  7.151, *p*<0.01. No effects were found for the control condition or SECPT. Analyses comparing the stress tasks to the control tasks revealed no differences.

### N-Back Task (NB)

Dependent measures on the N-Back Task included response time (Table 2), hit rate (Table 3), and sensitivity (d′; Table 4). Sensitivity is a composite index of hit rate and false alarm rate, which was calculated by subtracting the z-score of the false alarm rate from the z-score of the hit rate. Response time data reflect n  =  23, as one subject responded with a Hit Rate of 0 during 1 block of trials. Across all conditions, analyses revealed main effects of Load (all *p* <0.01), in which hit rate and d′ were greater in the 1-back and 2-back than 3-back loads, and response time was lower in the 1-back and 2-back compared to 3-back load. A main effect on Time on response time showed that response time decreased across the three iterations of the task (*p*s <0.05). A marginal Stress x Time interaction *F*(6,138)  =  1.884 (*p* <0.1) indicated that response times were higher immediately following the TSST (*p* <0.05) than 10- and 20 minutes after, and marginally lower immediately after the Control (*p* <0.1) than 10- and 20 minutes after, but no differences were found following the SECPT or MAT. No further effects were found for Stress, Time, or Stress x Time interactions (all *p*s > 0.28).

**Table 2:** N-Back Reaction Time (seconds) as a function of Stress and Load (means and SE) at baseline and 0-, 10- and 20-minutes post-stress.

Time	Load	TSST		SECPT		MAT		Control	
0 Min	1	723.63	(40.31)	722.18	(25.63)	701.57	(27.47)	705.97	(29.30)
	2	780.98	(50.28)	771.82	(32.34)	748.97	(29.34)	772.73	(47.10)
	3	793.38	(50.32)	763.38	(41.23)	737.55	(39.86)	760.52	(57.48)
10 Min	1	637.90	(25.24)	685.75	(20.99)	688.84	(28.68)	701.42	(29.54)
	2	721.31	(39.39)	774.79	(37.95)	716.03	(32.32)	779.72	(50.51)
	3	747.11	(39.65)	760.93	(52.05)	773.67	(34.57)	762.58	(43.66)
20 Min	1	667.04	(35.02)	670.79	(34.86)	681.82	(25.18)	701.25	(31.89)
	2	736.56	(44.25)	695.29	(30.24)	716.14	(42.37)	780.35	(44.84)
	3	738.01	(38.35)	716.71	(41.96)	717.72	(41.65)	779.68	(48.07)

No significant effects were found for Stress, Time, Load or any interactions.

**Table 3:** N-Back Hit Rate as a function of Stress and Load (means and SE) at baseline and 0-, 10- and 20-minutes post-stress.

Time	Load	TSST		SECPT		MAT		Control	
0 Min	1	0.77	(0.04)	0.78	(0.04)	0.76	(0.05)	0.72	(0.05)
	2	0.68	(0.04)	0.69	(0.04)	0.64	(0.04)	0.64	(0.04)
	3	0.49	(0.03)	0.47	(0.04)	0.53	(0.04)	0.48	(0.03)
10 Min	1	0.73	(0.05)	0.76	(0.04)	0.76	(0.04)	0.71	(0.05)
	2	0.69	(0.04)	0.66	(0.04)	0.67	(0.04)	0.67	(0.04)
	3	0.49	(0.04)	0.48	(0.04)	0.50	(0.04)	0.48	(0.03)
20 Min	1	0.74	(0.04)	0.73	(0.05)	0.75	(0.05)	0.68	(0.06)
	2	0.65	(0.05)	0.66	(0.05)	0.66	(0.04)	0.68	(0.04)
	3	0.51	(0.04)	0.49	(0.04)	0.45	(0.04)	0.51	(0.03)

No significant effects were found for Stress, Time, Load or any interactions.

**Table 4:** N-Back Sensitivity (*d*′) as a function of Stress and Load (means and SE) at baseline and 0-, 10- and 20-minutes post-stress.

Time	Load	TSST		SECPT		MAT		Control	
0 Min	1	0.90	(0.14)	0.99	(0.18)	0.91	(0.19)	0.73	(0.19)
	2	0.55	(0.14)	0.63	(0.16)	0.42	(0.14)	0.45	(0.14)
	3	−0.03	(0.10)	−0.08	(0.10)	0.11	(0.12)	−0.02	(0.12)
10 Min	1	0.80	(0.18)	0.86	(0.17)	0.89	(0.17)	0.76	(0.19)
	2	0.63	(0.15)	0.47	(0.12)	0.49	(0.13)	0.53	(0.14)
	3	−0.01	(0.13)	−0.04	(0.11)	0.01	(0.10)	−0.02	(0.11)
20 Min	1	0.89	(0.18)	0.78	(0.18)	0.88	(0.19)	0.61	(0.21)
	2	0.51	(0.16)	0.54	(0.16)	0.50	(0.14)	0.57	(0.14)
	3	0.01	(0.13)	0.00	(0.12)	−0.14	(0.13)	0.04	(0.10)

No significant effects were found for Stress, Time, Load or any interactions.

### Physiological Measures

#### Heart Rate

**Figure 3:**
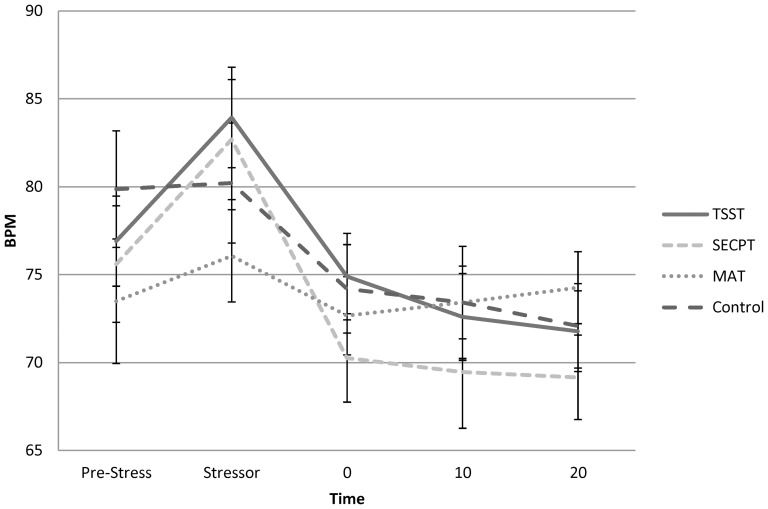
Heart rate. Heart rate as a function of stress condition and time (means and standard errors) pre-stress, during the stressor, and at 0, 10, and 20-minutes post-stress. Across all Stress conditions, heart rate was higher than baseline during the Stress tasks, and lower than baseline at all time-points post-stress (*p*<0.01). Additionally, heart rate higher than baseline during the TSST and lower than baseline 10-30 min post-TSST (*p*<0.01). Heart rate was higher than baseline during the SECPT and lower than baseline at all time-points post-SECPT (*p*<0.01). Heart rate was lower than baseline during all time points post-Control task *F*(4,92)  =  9.121, *p*<0.01. Heart rate did not change over time during the MAT.

Results revealed a main effect of Time, *F*(4,92)  =  43.729, *p*<0.01, in which heart rate was significantly higher than baseline during the stress induction, and lower than baseline at all time-points post-stress (Fig. 3). A Stress x Time interaction *F*(12,276)  =  4.624, *p*<0.01 showed a main effect of Time in the TSST, SECPT, and Control conditions, but not the MAT. In the TSST, heart rate was higher than baseline during the stress induction and lower than baseline 10-30 min post-stress *F*(4,92)  =  29.526, *p*<0.01 (no effects found 0-10 min post-stress). In the SECPT, heart rate was higher than baseline during the stress induction and lower than baseline at all time-points post-stress *F*(4,92)  =  31.379, *p*<0.01. During the control condition, heart rate was lower than baseline during all time points post-stress *F*(4,92)  =  9.121, *p*<0.01.

#### Salivary Cortisol

**Figure 4:**
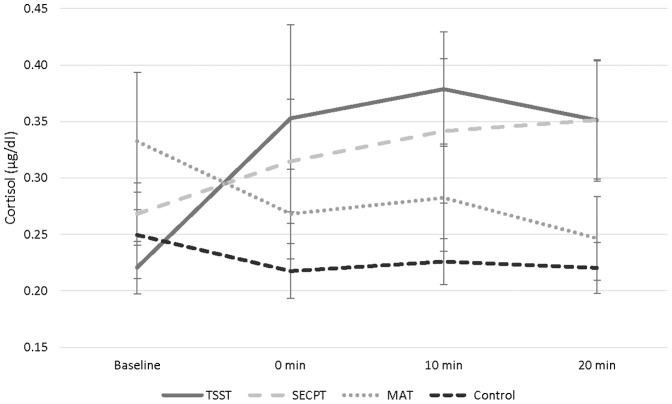
Salivary cortisol concentration. Salivary cortisol as a function of stress condition and time (means and standard errors) pre-stress and at 0-, 10-, and 20-minutes post-stress. Although ANOVAs were performed on log-transformed data, the figure shows raw data. Cortisol levels were higher than baseline at all time-points following the TSST (*p*<0.01), but not the SECPT, MAT or control task (all *p*s>.08). Additionally, cortisol levels were higher during the TSST and SECPT conditions 20-minutes post-stress (*p*<0.05) but not during the the MAT or for any stressors pre-stress, or immediately or 10 minutes post-stress (all *p*s>.06).

No baseline differences between the four Stress conditions were found (all *ps*>.35). Analyses of salivary cortisol revealed a Stress x Time interaction *F*(9,162)  =  3.894, *p*<0.01, in which cortisol levels were higher than baseline at all time-points post-stressor following the TSST *F*(3,54)  =  4.921, *p*<0.01 (Fig. 4). No differences were found for the SECPT, MAT or control task (all *p*s>.08). Analyses comparing the stress tasks to the control tasks at each time point showed that cortisol levels were higher in the TSST and SECPT conditions 20-minutes post-stress *F*(3,57) = 6.203, *p*<0.05. No differences were found for the MAT or for any stressors pre-stress, or immediately or 10 minutes post-stress (all *p*s>.06).

#### Salivary Alpha-Amylase (AA)

Analysis of baseline differences between the four Stress conditions yielded a main effect of Stress *F*(3,69)  =  3.594, *p* <0.05. sAA was higher before the TSST than Control (*p* <.01) and marginally higher before the SECPT than Control (*p* <0.1). No effects were found for Stress or Time, nor Stress x Time interactions (Table 5).

**Table 5:** Salivary alpha amylase (AA) as a function of stress (means and SE) at baseline and 0-, 10- and 20-minutes post-stress.

	TSST		SECPT		MAT		Control	
Pre-Stress	31.15	(5.04)	30.46	(8.38)	24.34	(4.37)	18.31	(2.64)
0 min	29.64	(4.97)	28.16	(5.23)	33.08	(5.70)	29.16	(4.70)
10 min	28.20	(4.66)	28.07	(6.45)	24.88	(4.10)	25.20	(3.24)
20 min	26.83	(4.39)	27.63	(5.94)	22.97	(3.78)	22.08	(3.74)

Although ANOVAs were performed on log-transformed data, the figure shows raw data. No significant effects were found for Stress or Time, nor Stress x Time interactions.

## Discussion

We compared the magnitude and duration of three experimental psychosocial stress induction paradigms on physiology, mood, and cognition. As hypothesized, the Trier Social Stress Test (TSST) exerted the most robust mood and physiological effects, followed by the Socially Evaluative Cold Pressor Task (SECPT). Specifically, the TSST increased feelings of tension, total mood disturbance, salivary cortisol and heart rate as well as reduced feelings of fatigue. With the exception of heart rate, which declined beginning 10-minutes post-stress, all TSST effects were evident immediately post-stress as well as 10- and 20-minutes after stress cessation. The SECPT increased heart rate as well as decreased feelings of fatigue. Effects of the Mental Arithmetic Task (MAT) were limited to increasing total mood disturbance. Comparisons of the stress tasks to the control task showed that relative to the control, the TSST resulted in elevated feelings of tension immediately post-stress, and both the TSST and SECPT reduced feelings of fatigue and increased cortisol levels 20-minutes post-stress. The stress response duration is a key to planning a study that utilizes an experimental stressor, as any dependent variable must be measured before the stress response subsides. The TSST would allow a longer window to administer tasks of interest.

There are several explanations as to why the MAT evoked a smaller stress response than the TSST and SECPT. First, the socioevaluative component of the TSST and SECPT are thought to contribute to the stress response [10], [36], [37]. Because the socially evaluative component of the MAT involves viewing another individuals' test score relative to one's own score rather than face-to-face contact with the evaluator, the MAT may invoke less socially evaluative pressure. Second, individual differences in arithmetic ability may have left this task susceptible to heterogeneity of responses. Average (± SEM) scores on the control MAT task were 67.36±2.95 compared to 61.22±5.84on the stressful MAT, and the average difference score between the control and stressful task was only 3.22±3.99. Only 11 out of the 24 subjects showed a decrease in score between the control and stressful MAT. Previous research has shown the individual differences in math anxiety influences math performance and cortisol response [38]–[40]. We did not find additional effects when we restricted the analyses to individuals whose scores were lower in the stressful versus control MAT, but this could be due to low sample size in this group.

The influence of the TSST and SECPT on mood was consistent with previous studies [15], [18], [41]. The TSST increased salivary cortisol levels, a biomarker of HPA activation, and both the TSST and SECPT resulted in higher cortisol levels than the control task 20-minutes post-stress. Participants were asked to keep their arms in the water for 3 minutes, but most withdrew their arms earlier (mean ± SEM  =  74.0 ± 21.5 seconds). Although this duration is within the range of studies that used the cold pressor to assess pain tolerance [42], it is shorter than that observed in other studies finding cortisol effects of the SECPT [18], [19], [43] and may account for the null effects. Of course, obtaining institutional review approvals for forced submersion durations might prove difficult or impossible. Future studies using this task should measure pain and perceived stress, to determine whether pain sensation supersedes the stress effects of this task.

Contrary to our hypotheses, we did not find stress effects on salivary alpha-amylase (AA), a biomarker of autonomic nervous system activation. Although AA levels fluctuate throughout the day, with a steep decline within 30 minutes of waking followed by gradual increase throughout the day [44], this is not likely to account for our null findings, as all participants began their test sessions in the afternoon. The majority of previous studies found that experimentally-induced stress increased AA levels [23], [45], but some did not [27].

The literature on stress effects on working memory is mixed. Several neuroimaging studies assessed the influence of stress on working memory-related brain activation. Qin and colleagues (2009) found that stress impaired N-Back working memory performance and reduced working memory-related DLPFC activity. Conversely, Porcelli *et al.* (2008) found increased activation in the PFC, with no corresponding change in working memory performance [46]. A third study found that stress enhanced working memory performance and increased activity in the PFC and posterior parietal cortex (PPC) [47].

Task difficulty could account for the differential findings. For example, stress impaired performance on active tasks that require constant updating of information, including the OSPAN [18], [28] and N-Back [15], [48] but not passive tasks including the digit span [27], [29]. Mixed effects were found on the Sternberg paradigm, in that stress reduced response time but increased false alarms [17]. Alternatively, differences in N-Back stimuli may explain differences in results. Whereas our task used spatial stimuli (i.e. participants responded whether the stimulus was in the same location as the stimulus1-, 2-, or 3-back), other studies used digits [15], [48]. A large body of research has investigated whether the neural networks of working memory are modality-specific. Although the evidence is mixed, some reviews and meta-analyses report dissociation between spatial and non-spatial working memory, wherein spatial working memory relies on a dorsal information stream whereas visual working memory relies on a ventral stream [49]. However, no differences were found in prefrontal cortex activity between spatial and non-spatial working memory tasks, making stimuli differences an unlikely explanation for our failure to replicate stress-induced N-Back working memory impairment [49], [50].

### Limitations

A number of limitations apply to the current study, which may at least partially explain relatively weak findings relative to the stress literature. First, we did not find changes in AA in response to stress, which is inconsistent with extant findings [51]. The most likely explanation for such null results is baseline differences in AA levels between the four Stress conditions, which potentially masked stress effects, as AA levels were higher in individuals before beginning the TSST (*p* <.05) and marginally higher (*p* <.1) before the SECPT than before the Control task. Further, it has been suggested that the use of Salivettes, as in the current study, may not yield AA results as reliable as other methods such as spitting or passive drool [35].

Second, the Control Task was not a perfect control condition for all three stressors. The TSST, MAT and Control task lasted 20 minutes, whereas the SECPT had a maximum duration three minutes, and as previously discussed, the majority of the subjects did not reach this threshold. Thus the Control Task controlled for the duration of the TSST and MAT but not the SECPT. Further, participants were required to walk to an adjacent room to complete the TSST and SECPT, whereas those undergoing the MAT and Control task remained seated. The increased movement involved in the TSST and SECPT could have contributed to elevations physiological stress response [36]. In this way, the Control Task controlled for movement of the MAT but not TSST and SECPT. While acknowledging that the Control task was not an ideal control for all three Stress tasks, adding a specific Control task for each stressor would be impractical, in that it would nearly double the number of test sessions and thus potentiate practice effects.

Third, factors pertaining to participants' background which could also influence the stress response were not collected, including experience related to the tasks, such as public speaking and arithmetic, hours of sleep, and time of waking prior to testing [52], [53]. These variables were not tested in the present study and thus not included as covariates, potentially contributing to the relatively high within-group variability.

Finally, our sample of 24 individuals included 7 males and 17 females, meaning assessing potential gender differences in stress-induces changes to working memory was infeasible. We feel that our total sample size was sufficient, given that we employed a repeated measures design, whereas previous studies utilizing larger samples assessed between-subjects differences between stress and control groups [15], [48], [54], [55]. Further, our sample size is in line with other studies assessing stress effects on memory that have used repeated measures designs [46], [48], [56], [57], even those that have used between measures designs [28]. That said, the small number of males within the sample precluded any analysis of gender differences. Cortisol levels following stress were generally higher in males than females [58], [59] and cortisol responses were inversely associated with memory performance in males but not females [60]. Subsequent studies should utilize a larger sample size, particularly one large enough to examine potential gender differences.

### Conclusions

Comparison of the TSST, SECPT and MAT task indicates that the TSST and SECPT are likely the most effective methods of experimentally inducing acute stress, with the TSST proving the most robust and reliable. Together with previous studies, our data suggests that the TSST and SECPT impairs mood and increases HPA axis activity. The SECPT and MAT have the logistical advantage of requiring fewer investigators. Additionally, the MAT would enable investigators to run multiple subjects at once. However, participants are allowed to withdraw their arms from the cold water in SECPT when they wish, and many do so soon after it becomes uncomfortable. Therefore the physiological and mood effects of this task are generally limited to the duration of the stressor, thus reducing its utility in research requiring more sustained stress response.

Similarly, individual variability in arithmetic performance, and likely math anxiety, contributes to inconsistent effects of MAT task. Although the MAT did impair some aspects of mood, it had no influence on HPA or autonomic activity. Perhaps limiting participants to those with high math anxiety would increase the efficacy of the MAT task as an experimental stressor. However the task is not effective in a random cross-section of young adults. In conclusion, the present data support the continued use of the TSST as the prototypical experimental stressor, particularly relative to the SECPT and MAT.
